# Development of a goal setting and goal management system: Intervention Mapping

**DOI:** 10.3389/fresc.2023.1274191

**Published:** 2024-01-08

**Authors:** Eunyoung Kang, Erin R. Foster

**Affiliations:** ^1^ Program in Occupational Therapy, Washington University School of Medicine, St. Louis, MO, United States; ^2^ Department of Neurology, Washington University School of Medicine, St. Louis, MO, United States; ^3^ Department of Psychiatry, Washington University School of Medicine, St. Louis, MO, United States

**Keywords:** goals, patient care planning, intervention mapping, chronic conditions, occupational therapy, community-based participatory research, translational research

## Abstract

**Background:**

Although goal setting and goal management (GSGM) is a key component of chronic disease management and rehabilitation practice, there is currently no widely used evidence-based intervention system available. This paper describes the theoretical underpinnings and development of a new intervention called MyGoals. MyGoals is designed to guide occupational therapy (OT) practitioners to implement theory-based, client-engaged GSGM for adults with chronic conditions in community-based OT rehabilitation settings.

**Methods:**

We first developed a planning team with two adults with chronic conditions, two clinicians, and two researchers. As a collaborative team, we co-developed MyGoals by following Intervention Mapping (IM) steps 1–4 and incorporating community-based participatory research principles to ensure equitable, ecologically valid, and effective intervention development. In the first step, the planning team conducted a discussion-based needs assessment and a systematic review of current GSGM practice to develop a logic model of the problem. In the second step, the planning team identified performance objectives, intervention target personal determinants, and change objectives, and created a logic model of change and matrics of change objectives. In the third step, the planning team designed MyGoals. Lastly, in the fourth step, the planning team produced, pilot-tested, and refined MyGoals.

**Results:**

The ultimate goal of the MyGoals intervention is to enable clients to achieve personally meaningful rehabilitation goals. The planning team identified four target determinants (e.g., self-efficacy), six intervention activities (e.g., *Education, Reflection, Find My Goals, Make My Goals, Make My Plans, My Progress*), eight performance objectives (e.g., List potential goals), and 26 change objectives (e.g., Understand the importance of GSGM). Two pilot tests indicated that MyGoals is feasible for clients and identified areas for improvement. Based on the feedback, minor refinements were made to the MyGoals intervention materials.

**Conclusions:**

We completed rigorous and collaborative IM to develop MyGoals. Establishing the theoretical and developmental foundation for MyGoals sets the groundwork for high-quality, evidence-based GSGM. Future studies on effectiveness and implementation are necessary to refine, translate, and scale MyGoals in rehabilitation practice.

## Introduction

Goal setting and goal management (GSGM) is a fundamental rehabilitation practice in which clients and clinicians collaboratively establish goals, develop plans, evaluate goal progress and achievement, and adjust goals and plans (1–4). The goal setting process includes evaluating clients’ values, assessing current and desired functioning, setting goals, and making plans. Meanwhile, the goal management process involves evaluating goal progress and achievement, as well as adjusting goals and plans as needed. Throughout GSGM, clients and clinicians develop an understanding of client-related factors (e.g., clients’ needs, health conditions, and environment), enhance their working relationship, and make shared goals and plans (2, 5–7). The established goals and plans provide clients and clinicians with a mutual direction for treatment, and thus can promote person-centered rehabilitation implementation (5).

While there are several frameworks and approaches that emphasize a person-centered, collaborative approach, such as SMART rehabilitation goals and MEANING, current GSGM in rehabilitation practice remains suboptimal, primarily due to two major practice gaps (8–13). These include limited implementation of theory-based intervention components and poor client engagement in the intervention (8). Most interventions do not fully incorporate all essential theory-based GSGM components (8). Components related to coping planning, goal monitoring, goal evaluation, and goal adjustment are particularly under-utilized in current practice even though they are likely as important as other frequently used components such as goal formulation (8).

Achieving active client engagement in GSGM is another major challenge in practice (8, 14, 15). Active client engagement in GSGM may promote better outcomes such as a better sense of ownership of rehabilitation care, quality of life, self-reported emotional status, and self-efficacy (5, 16). However, current practice does not facilitate active client engagement (8). Clinicians often fail to enable clients to participate actively in GSGM (17, 18). This is not because clinicians do not have the knowledge, but rather because they have difficulty translating their knowledge into practice (17). We need a new practical and effective system to address these research-practice gaps by guiding clinicians to implement high-quality GSGM (8, 18–20).

Developing evidence-based interventions requires a systematic approach that includes a review of literature and theories, collaboration with community partners such as clients and practitioners, implementation strategy development, and intervention adaptation and evaluation (21). In rehabilitation, especially in occupational therapy (OT), there have been challenges in establishing ecologically valid evidence-based interventions. This is due to inadequate identification and description of intervention components and mechanisms, as well as insufficient consideration of the preferences and needs of target clients and practitioners (22–25).

To address these limitations, the use of a theory-based, collaborative approach such as Intervention Mapping (IM) and community-based participatory research principles is actively advocated for the development of interventions (21, 24–27). A growing number of studies have began to utilize IM with community-based participatory research in developing interventions across various fields and contexts (28–30). However, despite their potential, there are few GSGM interventions that utilize these approaches in their development. To address this gap, we developed a new system to guide theory-based, client-engaged GSGM, called MyGoals. MyGoals is designed to support practitioners to implement comprehensive, client-engaging GSGM for adults with chronic conditions in community-based rehabilitation using six intervention activities.

In this paper, we describe the developmental process and detail the theoretical background of MyGoals using IM in collaboration with two adults with chronic conditions, two clinicians, and the research team (21, 27). We elaborate on MyGoals’ logic model of the problem, logic model of change, matrix of change, mechanisms of action, intervention targets, active ingredients, production, and refinement. In its current initial developmental stage, MyGoals is designed primarily for implementation in an OT context. However, the long-term goal of this development is to translate this new intervention into broader rehabilitation practice, with additional work to ensure its clinical utility and generalizability.

## Materials and methods

### MyGoals Intervention Mapping (IM) conceptual model

**Figure 1:**
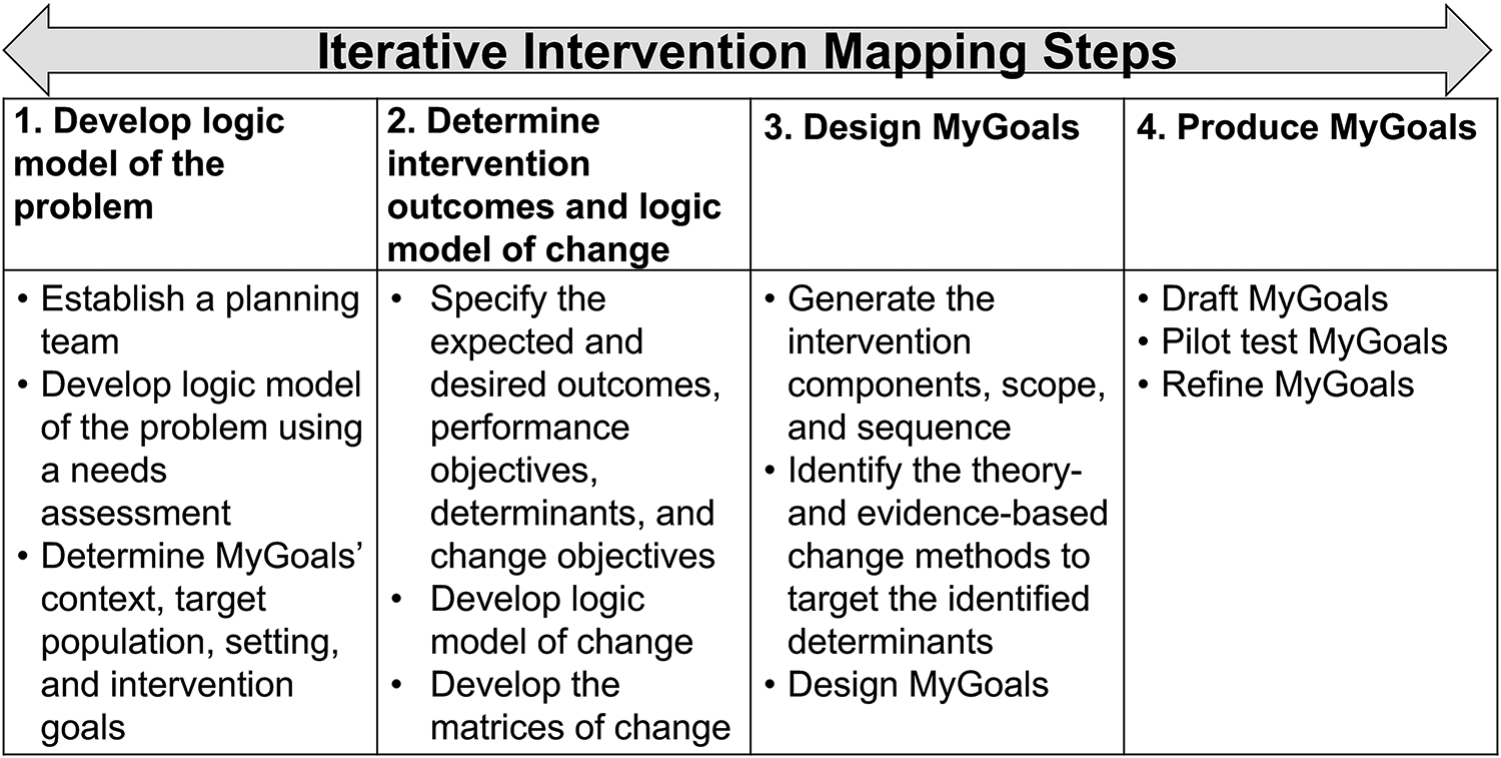
The working conceptual model of MyGoals.

IM is a set of six iterative tasks to guide the identification of behavior determinants, mechanisms of action, intervention strategies, active intervention ingredients, and outcomes to develop theory-based interventions (21). IM includes the following tasks: (1) Developing a logic model of the problem to identify the target health problems and understand the behavioral and environmental factors, as well as personal determinants (e.g., self-efficacy, knowledge), that lead to the identified health problem, (2) Creating a logic model of change to determine the desired health outcomes and describe which behavioral and environmental factors and personal determinants need to be addressed to achieve these outcomes, (3) Designing the intervention, which includes determining its dose, components, delivery methods, materials, and other aspects, (4) Producing the intervention, (5) Planning intervention implementation by developing strategies to deliver the intervention, and (6) Evaluating the intervention based on the logic model of change (21). Theories, models, and frameworks play central roles across IM steps to develop theory-based interventions. Each IM step provides a guide on how to use theories, models, and frameworks to determine intervention target behaviors, goals, components, approaches, mechanisms of action, and others. The working conceptual model of MyGoals IM is illustrated in Figure 1 and detailed in the below paragraphs. The findings from IM steps 5 and 6 are published elsewhere (31–33).

#### IM step 1. Logic model of the problem

The first step involves establishing a planning team and developing a logic model of the problem (21). The planning team was developed, including two adults with chronic conditions (MyGoals target clients), two clinicians, and the research team. Although the size of the planning team is not particularly large, we chose the current size to gain in-depth perspectives from each individual member. Furthermore, since we planned to incorporate extensive literature and theories, this team size was deemed appropriate, especially when considering logistical limitations. For instance, a larger team size would require more time commitment from members to accommodate all perspectives.

Given the small number of planning team members, we decided not to report detailed demographic information to avoid the potential identification of any individual. The median age of two client participants was 73.5 years old (SD = 10.6), and they were male and female. All planning team members self-identified as non-Hispanic or Latino and either Asian, Black or African American, or White. Every member had at least a high school degree. The diagnoses among client members included Parkinson's disease, cancer, and diabetes. Clinicians were OT practitioners employed in community-based practice settings. The median years of professional working experience for the clinicians was 10 (SD = 4.2). We completed a discussion-based needs assessment and a systematic review of current GSGM practice to identify health problems, determinants, behavior problems, and essential GSGM intervention components (8). Based on these findings, the team developed the logic model of the problem and clarification of MyGoals intervention context, target population, setting, and intervention goals.

#### IM step 2. Logic model of change

The second step involves developing the logic model of change with the expected behavior outcomes, performance objectives, personal determinants, and matrices of change objectives by using the IM guided questions (e.g., “What do clients need to do to achieve their personally meaningful goals?”) (21). We drafted performance objectives and compared them with essential GSGM components that we identified from the aforementioned systematic review (8) to determine how these objectives can be incorporated into MyGoals. Then we reviewed the literature, brainstormed potential determinants, and selected key determinants for each performance objective. We also developed change objectives to define desired changes at the personal determinant level. Combining all findings from the second step, we developed the MyGoals Logic Model of Change and the matrices of change objectives.

#### IM step 3. Intervention theories, approaches, and design

The third step involves generating the intervention components, scope, and sequence, selecting theory- and evidence-based behavior change methods, and designing a practical intervention that satisfies the parameters of effectiveness (21). Based on the findings from the previous steps, we determined the intervention components, scope, and sequence and theory- and evidence-based change methods to target the identified determinants using Social Cognitive Theory (34–37), Self-Determination Theory (38, 39), The Theory Of Intentional Action Control (40–42), and A Taxonomy Of Behaviour Change Methods (43).

#### IM step 4. Intervention manual, manual, instructions, scripts, supplements

The fourth step involves MyGoals production, evaluation, and refinement (21). We drafted the MyGoals manual, instructions, scripts, supplements, and client worksheets (see example in Supplementary File S1). The materials are designed to provide structure and support and to enable clinicians to implement MyGoals in their practice without significant modifications; however, they are not meant to be prescriptive. Rather, MyGoals is designed with flexibility in its delivery (e.g., time allocated for each activity, streamlining of activities) depending on practice setting, clients, etc.

Two rounds of in-person MyGoals pilot evaluations were completed with the planning team members. The OT-client planning team dyad completed two in-person sessions (1st session: complete *MyGoals activity 1. Education*—*5. Make My Plan* and 2nd session: Complete *MyGoals activity 6. My Progress*). After the pilot evaluation, the planning team discussed and identified areas for improvement, and then revised the developed materials.

## Results

### IM step 1. Logic model of the problem

**Figure 2:**
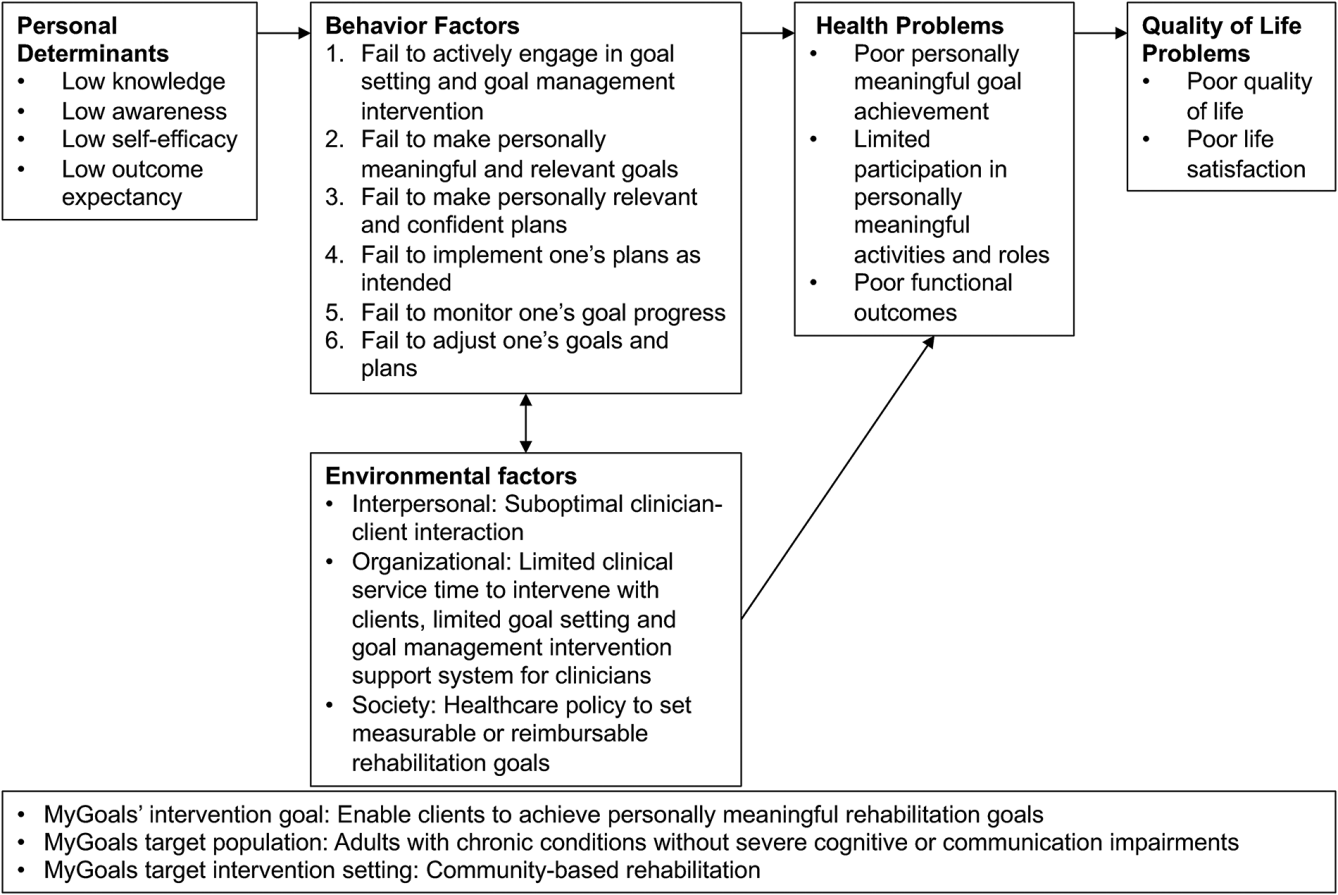
MyGoals Logic Model of Problem.

The overall goal of the MyGoals intervention was identified as *enabling clients to achieve personally meaningful rehabilitation goals*. The target population for MyGoals was adults with chronic conditions who do not have severe cognitive and communication impairments, and the setting was community-based rehabilitation. We specified poor goal achievement as a primary health issue that MyGoals was designed to address, as illustrated in the MyGoals Logic Model of the Problem (Figure 2). Subsequently, we identified specific behavioral and environmental factors, as well as personal determinants, that correlate with poor goal achievement within the target population.

Self-efficacy is a key determinant that has shown positive associations with goal-directed behavior (44, 45). Social Cognitive Theory suggests that people with lower self-efficacy are less likely to actively commit and persist in their goals (36, 37, 46). Outcome expectancy is another important determinant from Social Cognitive Theory that can drive goal-directed behavior (34, 35, 47). People evaluate the potential positive and negative outcomes of their goals and then develop intentions to act or not act on their goals (34, 35, 47). Thus, when people do not have an adequate level of positive outcome expectancy for their goals, they are less likely to take goal-directed behavior (48, 49). Inadequate knowledge of GSGM concepts and expected roles during GSGM can lead to poor engagement in the intervention or limit goal-directed behaviors, which can result in poor goal achievement (50). Lastly, poor awareness about oneself can hinder the development of personally meaningful goals and goal achievement (51–53).

Through our systematic review of the current GSGM literature, we identified 12 essential components of GSGM: education, reflection, goal exploration, goal formulation, action planning formulation, goal/plan barrier identification, goal/plan facilitator identification, goal/plan confidence evaluation, coping planning formulation, evaluation, self-evaluation, and goal/plan adjustment (8). Details about these components are outlined in the Step 3 results below.

### IM step 2. Logic model of change

**Figure 3:**
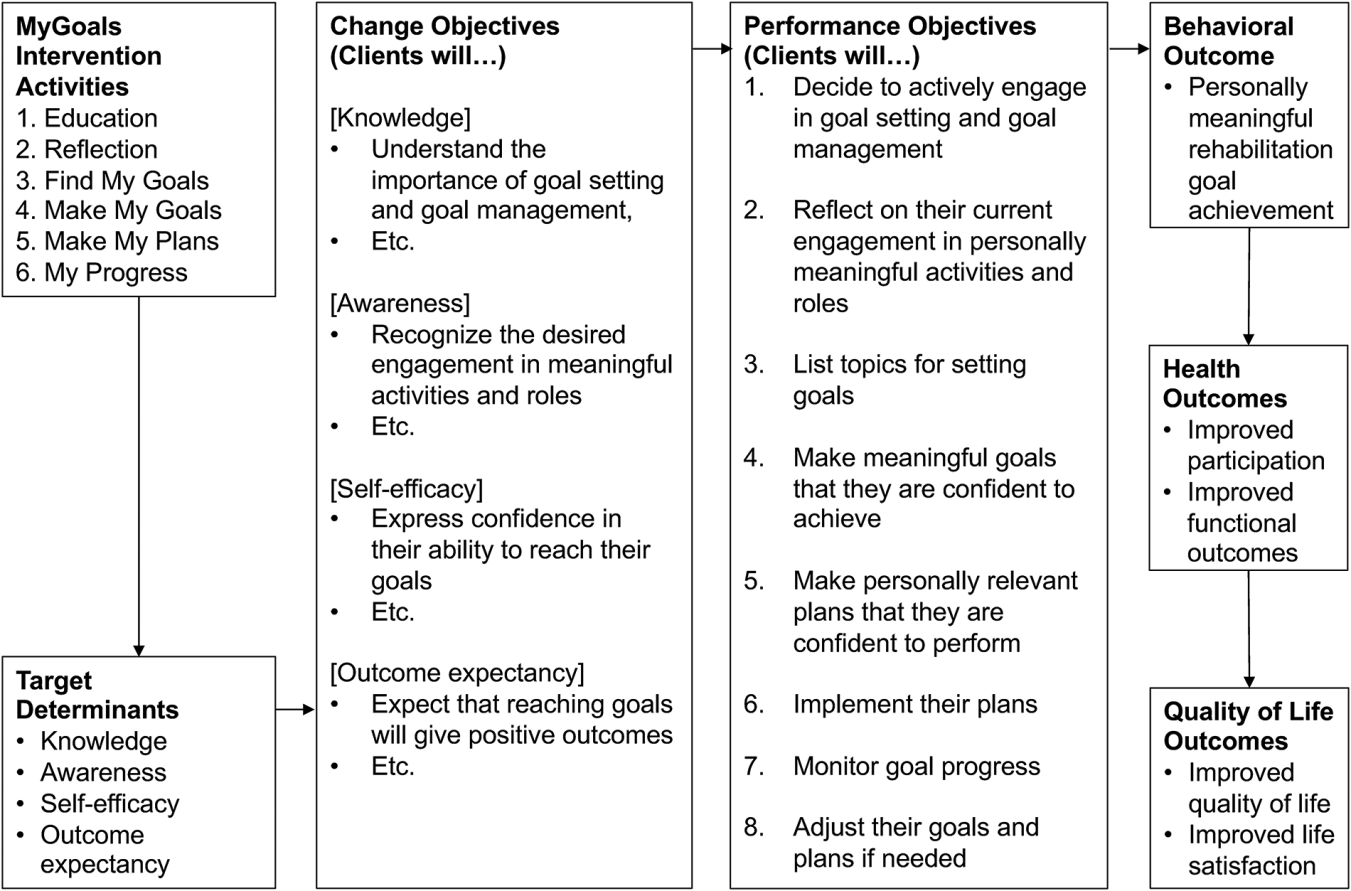
MyGoals Logic Model of Change.

We developed the MyGoals Logic Model of Change (Figure 3) and the matrices of change objectives (Table 1). In Figure 3, we outlined the performance and change objectives necessary for clients to achieve the identified behavioral outcome of MyGoals (i.e., personally meaningful rehabilitation goal achievement). Further details about change and performance objectives, as well as determinants (intervention targets) of MyGoals, are described in Table 1.

**Table 1:** MyGoals matrices of change objectives.

MyGoals activities	Performance objectives (Client will…)	Change objectives (Client will…)			
		Knowledge	Awareness	Self-efficacy	Outcome expectancy
1. Education	Decide to actively engage in GSGM	1.1. Understand the importance of GSGM 1.2. Understand one's expected role during GSGM	NA	NA	NA
	Mechanisms of action	Discussion, individualization, participation	NA	NA	NA
2. Reflection	Reflect on their current engagement in personally meaningful activities and roles	NA	2.1. Recognize their current engagement in meaningful daily activities and roles, health, and environment	NA	NA
	Mechanisms of action	NA	Individualization, participation, self-reevaluation	NA	NA
3. Find my goals	List topics for setting goals	NA	3.1. Recognize their desired engagement in meaningful activities and roles	NA	NA
	Mechanisms of action	NA	Individualization, participation, self-reevaluation	NA	NA
4. Make my goals	Make goals that they feel meaningful and confident to achieve	4.1. Understand the concepts of life goal, goal, and building block goal	4.2. Recognize their life goal, goals, and building block goals	4.3. Express confidence in their ability to reach their goals	4.4. Expect that reaching their goals will give positive outcomes
	Mechanisms of action	Advance organizers, discussion, elaboration, individualization, participation	Participation, individualization	Goal setting, individualization, participation, public commitment, set graded tasks	Elaboration, individualization, participation, self-reevaluation
5. Make my plans	Make plans that they feel personally relevant and confident to perform	5.1. Understand the if-then plan concept 5.2. Understand the concepts of barriers and facilitators 5.3. Understand the concept of planned action	5.4. Recognize their barriers and facilitators to reaching goals 5.5. Recognize their planned action	5.6. Express confidence in their ability to perform plans	5.7. Expect that reaching plans will give positive outcomes
	Mechanisms of action	Discussion, individualization, participation	Individualization, participation, self-reevaluation	Goal setting, implementation intention, individualization, participation, planning coping responses, public commitment, set graded tasks, verbal persuasion	Elaboration, individualization, participation, self-reevaluation
6. My progress	Implement their plans	6.1. List their plans	6.2. Recognize their current situation and plans	6.3. Express confidence in their ability to perform plans	6.4. Expect that reaching goals or plans will give positive outcomes
	Mechanisms of action	Participation	Participation	Participation	Participation, self-reevaluation
	Monitor goal progress	6.5. Understand their expected roles during goal management	6.6. Recognize their goal progress	6.7. Express confidence in their ability to monitor goals and plans	NA
	Mechanisms of action	Discussion, individualization, participation	Individualization, participation, self-reevaluation	Enactive mastery experiences, feedback, improving physical and emotional states, individualization, participation, self-monitoring of behavior	NA
	Adjust their goals and plans if needed	6.8. Understand that they can adjust goals and plans if needed	6.9. Recognize their current situation, goal, and plans	6.10. Express confidence in their ability to adjust goals and plans	6. 11. Expect that adjusting goals or plans will give positive outcomes
	Mechanisms of action	Discussion, individualization, participation	Individualization, participation, self-reevaluation	Feedback, goal setting, individualization, participation, verbal persuasion	Individualization, participation, self-reevaluation

For instance, to achieve the performance objective of deciding to actively engage in GSGM, clients should gain knowledge (i.e., determinant). Specifically, they need to achieve two knowledge-related change objectives including *understanding the importance of GSGM* and *understanding one’s expected role during GSGM*. By achieving these two change objectives, clients become more likely to achieve the performance objective.

### IM step 3. Intervention theories, approaches, and design

To address all 12 identified GSGM components, we developed six MyGoals activities (Table 2). Table 2 describes how each MyGoals activity addresses the GSGM components idenfied in step 1 and its corresponding objectives. After completing step 3, we iteratively refined the matrics of change objectives by using the six MyGoals activities as outlined in Table 1.

**Table 2:** MyGoals intervention activities, the GSGM component(s) they address, and their objectives.

MyGoals intervention activities (components addressed^a^)	MyGoals intervention activity objectives
Activity 1. Education (Education)	• Educate the basic concepts of GSGM • Educate on clients’ expected roles during the intervention
Activity 2. Reflection (Reflection)	• Guide reflection on one's current engagement in personally meaningful activities and roles, health status, or behaviors
Activity 3. Find my goals (Goal topic identification)	• Guide identification of goals that clients want to start, learn, do more easily or efficiently, etc.
Activity 4. Make my goals (Goal formulation)	• Guide life goal, goal, building block goal formulation • Guide evaluation of self-efficacy and outcome expectancy levels of the developed goals • Educate and discuss the benefits of using life goals, goals, building block goals • Educate and discuss clients’ health conditions using a person-centered approach
Activity 5. Make my plans (Action plan formulation, goal/plan barrier identification, goal/plan facilitator identification, goal/plan confidence assessment, coping plan formulation)	• Guide barrier, facilitator, and planned behavior identification to make if (when)—then plans • Guide if (when)—then plan formulation • Guide self-efficacy evaluation for the formulated plans
Activity 6. My progress (Self-evaluation, professional goal/plan progress evaluation, goal/plan adjustment)	• Educate on client's expected roles during goal management • Guide self-evaluation of goal progress and satisfaction • Discuss goal progress and goal/plan adjustment

^a^ These GSGM components were identified from our systematic review (8) and existing literature (54).

We identified theory- and evidence-based change methods to target the identified determinants using Social Cognitive Theory (34–37), Self-Determination Theory (38, 39), the theory of intentional action control (40–42), and the taxonomy of behaviour change methods as described in Supplementary File S2 (43). We further took into consideration the parameters for effectiveness based on Kok et al. (43) to activate the identified mechanisms of action in MyGoals as described in Table 3 and Supplementary File S2. For instance, as described in Table 3, *participation* (in the intervention) is known as an effective mechanism of action when clients want to and have the ability to participate in interventions and clinicians are willing to collaborate with clients as co-partners (43). Therefore, MyGoals provides clinicians with the educational resources to understand the importance of active client engagement and encourages them to foster a more open client-clinician partnership during interventions. MyGoals also incorporates various intervention strategies, such as open-ended questions, into the manual to support clinicians in promoting active client engagement. Additional details about this and other examples of mechanisms of action and parameters in MyGoals are provided in Table 3 and Supplementary File S2.

**Table 3:** Example mechanisms of action and parameters for effectiveness incorporated in MyGoals.

Mechanisms (applied parameters of effectiveness)	How this parameter is incorporated in MyGoals [MyGoals activity number(s) or OT education]
Participation (Clinicians’ willingness to accept clients as active partners in their care; clients with motivation and skills)	• Educate clinicians about the importance of active client engagement (OT education) • Ask open-ended questions and develop easy to participate activities using lay language (1–6) • Encourage clients to actively participate in MyGoals activities using verbal education and read out loud the MyGoals summary sheet (1, 6) • Guide clients to reflect on their current engagement in activities and their health and environment and share their reflections (2) • Guide clients to come up with and write down activities and roles they want to work on using the Find My Goals Sheet (3) • Guide clients to come up with and write down personally meaningful goals with high confidence and positive outcome expectancy using the make my goals sheet and guide them to realize potential positive outcomes of the developed goals (4) • Guide clients to come up with and write down their facilitators, barriers, and planned actions using my plan and progress sheet, guide clients to develop personally relevant and confident plans, with high positive outcome expectancy, and guide clients to realize potential positive outcomes of the developed plans (5) • Guide clients to monitor their goal progress using the my plan and progress sheet, guide clients to adjust their goals and/or plans to develop personally meaningful goals and relevant plans with high positive outcome expectancy (6)

### IM step 4. Intervention manual, manual, instructions, scripts, supplements

We drafted the MyGoals manual, instructions, scripts, supplements, and client worksheets. Supplementary File S1 includes an example of a MyGoals activity, Activity 4: Make My Goals, and a Case Study is included below to illustrate the entire process. For instance, *Activity 4. Make My Goals* is designed to enable clinicians to guide clients to first develop participation-based goals that are intrinsicly motivating as suggested by Self-Determination Theory (38, 39). Then clients make goals related to activity, health conditions, and body functions & structures that will support achievement of their participation-related goals. *Activity 3. Make My Plans* is designed to help clinicians guide clients to identify their goal facilitators and barriers. Identifying barriers and faciltators can help the client improve their awareness about their capacity and available resources to improve their competency as suggested by Self-Determination Theory (38, 39) and self-efficacy to achieve their goals as suggested by Social Cognitive Theory (34–37). Based on the identified factors, clients are guided to develop if-then plans informed by the theory of intentional action control (40–42).

The materials are designed to provide structure and support and to enable clinicians to implement MyGoals in their practice without significant modifications; however, they are not meant to be prescriptive. Rather, MyGoals is designed with flexibility in its delivery (e.g., time allocated for each activity, streamlining of activities) depending on practice setting, clients, etc.

After the pilot evaluation, we found that most parts of MyGoals seemed feasible from both clients’ and clinicians’ perspectives. We made several minor revisions based on our pilot testing. We added detailed explanations about different goal types to facilitate goal formulation. We also included a written summary of basic GSGM concepts and clients’ expected roles during the intervention. We made minor revisions to the scripts and instructions on the client worksheets to ensure that MyGoals uses lay language to facilitate the interactive client-OT conversation and client engagement throughout the MyGoals intervention.

### Case study

We provide a brief case study to illustrate the application of MyGoals.

Kasey is a 55-year-old librarian who had a stroke one year ago, which resulted in mild right upper extremity impairment, mild depressive symptoms, and mild cognitive impairment. After intense inpatient rehabilitation, Kasey made a full recovery in her upper extremity and now lives independently in an apartment with their dog, Bella, and has returned to work. Prior to her stroke, Kasey had a physically and socially active lifestyle and enjoyed weekend hikes with Bella. However, since returning home and to work, Kasey notes moderate fatigue and a decrease in physical activity throughout the day, especially after work. She has stopped taking Bella for walks and meeting up with friends regularly. Kasey has also noticed mild forgetfulness, difficulty managing her daily routine, and difficulty focusing at work. In the past two weeks, Kasey has become concerned about whether she can continue to live independently at home, work, and stay healthy and has experienced increased feelings of depression and anxiety. Due to these concerns, Kasey was referred to a community-based OT for self-management.

• *Activity 1. Education:* Kasey learned about the importance of goal setting and goal management, as well as the importance of active engagement during the intervention, to make personally meaningful goals and realistic and relevant plans to achieve better participation and health. Kasey mentioned that she tends to feel overwhelmed when she needs to make a sudden decision or when things do not go as planned. Therefore, Kasey prefers to have a clear plan as well as a backup plan to better handle daily life activities.
• *Activity 2. Reflection:* Kasey reflected on her functions, including cognitive, emotional, and physical functions, as well as her engagement in meaningful activities. She identified exercise, walking her dog, being with friends, work, and reading as meaningful, enjoyable, and important activities. Among these, Kasey wanted to focus on exercise, walking the dog, and work during therapy.
• *Activity 3. Find My Goal:* Kasey made three potential goals: “exercise for 15 min every day,” “walk my dog,” and “work productively.” Kasey rated their importance as 9’s for the first and 7 for the last two (on a scale of 1–10, with higher scores indicating higher importance). Kasey decided to focus on the following goal for therapy: “walk my dog.”
• *Activity 4. Make My Goal:* Kasey chose to work on her goal of “walking my dog” because it helps her exercise, take care of Bella, and casually interact with friends and neighbors while walking. Kasey then specified the goal as follows: “I will walk Bella for 30 min every day after my breakfast.” She rated its importance as a 9 and positive outcome expectancy as a 10. Kasey also made a life goal of “having a physically and socially active life” and a building block goal of “better managing my energy and fatigue” to support achieving the goal. Kasey discussed how these goals were interrelated and could support better participation and health.
• *Activity 5. Make My Plan:* Kasey identified three barriers to achieving her goal of walking Bella every day—feeling tired, forgetting to walk Bella, and concern about neighborhood safety while walking alone. After identifying the barriers, the OT educated Kasey about general fatigue management and energy conservation strategies. Then, the OT and Kasey discussed together which specific strategies would work best for Kasey and how to personalize these for Kasey's preferences, needs, and life situations to make personally relevant and realistic plans. After the education and discussion, to overcome the first and second barriers, Kasey identified a facilitator: walking right after breakfast because it is the most energizing time for her, and because having breakfast is a stable part of her morning routine. To overcome the last barrier, Kasey identified another facilitator: walking in the neighborhood during busy morning hours to ensure many neighbors are around for safety. Kasey made an if(when)-then plan to achieve their goal and rated their confidence in reaching it as 9 out of 10. The plan is as follows: “When I finish my breakfast, then I will walk Bella for 30 min in my neighborhood.”
• *Activity 6. My Progresss:* The following week, Kasey returned to therapy and discussed with the OT practitioner about what helped and prevented her from carrying out her plan. Kasey said having a specific action plan helps her feel less overwhelmed because she knows what needs to be done and how. At the same time, choosing the time as “after breakfast” instead of an exact time gave Kasey some flexibility in managing her time but also helped ensure she would not forget to take Bella for a walk since it happened every day. Additionally, because Kasey knew that there would be many neighbors out during morning hours, she felt more comfortable walking outside alone. Kasey mentioned that she sometimes feel too tired to walk for 30 min, and when this happens, she tends to give up. Kasey said having backup plan for tired days can be helpful to at least walk a little bit without giving up the routine. Kasey rated her performance and satisfaction with her goal progress both as 9’s and discussed whether she wanted to adjust her goals and plans. Kasey wanted to keep the original plan and added a backup plan for days when she feels extra tired. Thus, Kasey made the following coping plan: “If I feel too tired to walk for 30 min, I will walk Bella as much as I can.” Kasey rated her confidence in reaching this plan as a 9 and continued to work on her goal by using two plans.

## Discussion

This paper provides the theoretical foundation, description, and development of a novel system designed to promote comprehensive and client-engaging GSGM. To our knowledge, this is first use of IM in developing GSGM for adults with chronic conditions in community-based rehabilitation. This offers important insights into the theoretical aspects and systematic development of MyGoals. It facilitates a thorough understanding of MyGoals and its implementation. Ultimatley, this work can produce quality evidence across contexts and contribute to the enhancement of high-quality GSGM practice implementation.

### Theoretical implications

Rehabilitation interventions often do not specify the theoretical processes that enable clients to achieve the desired intervention outcomes (23). Such practice hinders the understanding, evaluation, and replication of rehabilitation interventions and their mechanisms, and ultimately delays in establishing evidence-based interventions (22, 23). We have provided the theoretical background of MyGoals intervention target constructs, approaches, activities, mechanisms of action, and parameters for effectiveness. We specified why and how MyGoals’ theoretical constructs such as self-efficacy are incorporated into the intervention. We also laid the groundwork to establish theory-based mechanisms of action for MyGoals by developing hypothesized processes by which the intervention enables clients to achieve the change and performance objectives to reach the ultimate intervention goal. This specification provides a rationale for each activity as well as the overall intervention.

MyGoals can be seen as a natural synthesis and advancement of existing GSGM frameworks and approaches. In creating MyGoals, our objective was to develop a concrete, practical intervention system that would support practitioners in translating evidence-based interventions into practice more effectively. Existing frameworks and approaches offer overarching principles on how GSGM should be conducted or focus on specific GSGM intervention components (e.g., goal attainment scaling, which emphasize making and reviewing goals without delving into planning) (4, 10, 13, 55). On the other hand, MyGoals integrates traditional and foundational principles, such as person-centeredness, thorough assessments of clients’ values and functions, and enhancement of client-practitioner collaborations, into a tangible intervention system with a strong emphasis on clinical utility. Leveraging an implementation science framework and a community-engaged approach allowed us to transform the core principles of existing frameworks into a practical, theory-driven GSGM intervention that holistically addresses GSGM components, from education to goal adjustment.

Future studies can evaluate the mechanisms of action to disentangle how each activity works at the determinant-, performance-, or activity levels, not simply how MyGoals works as a whole. This will permit precise optimization of individual activities to improve the intervention's efficacy and effectiveness overall. The information provided in this paper will allow us to build more generalizable knowledge to establish evidence-based GSGM.

### Practice implications

MyGoals’ structured approach can support practitioners to easily implement theory-based GSGM components in practice and promote active client engagement during the intervention. In our needs assessment, clinicians expressed difficulties in using the existing GSGM tools in practice due to their lack of a structured approach and practical guidelines. MyGoals’ intervention activities, a detailed manual, clinician scripts, and a client worksheet can address this noted limitation in practice and support high-quality GSGM practice by providing concrete structured materials for practitioners.

We further enhanced the ecological validity of MyGoals by incorporating the perspectives of end-users including practitioners and clients into its development, which may improve its clinical utility. Indeed, we have conducted a feasibility study of MyGoals and found that clinicians consider MyGoals to be a feasible and promising tool to guide theory-based, client-engaging GSGM (33). Future studies will evaluate MyGoals’ effectiveness in supporting GSGM to promote person-centered OT rehabilitation and improve health in adults with chronic conditions. Additional work to validate MyGoals’ feasibility and effectiveness in other rehabilitation contexts is required.

### Limitations

The clinician and client team members had limited time to work on this project. Therefore, the research team needed to design the overall research process and prepare all meeting materials such as discussion questions, educational materials, articles, and logic model drafts. However, by promoting interactive discussion, we ensured the active engagement of all team members. In the future, partners should be provided with additional protected time and resources to allow them to take a more active role in the research. However, to do this we need to develop effective strategies to promote community-engaged research given that not all research studies have extensive resources, especially in early developmental stages.

## Conclusion

We provide in-depth information on the development of a new system, MyGoals, to support high-quality theory-based GSGM intervention implementation. IM successfully guided us in developing MyGoals through collaboration with clients and clinicians. Our systematic and theory-based intervention development and reporting will help other rehabilitation scientists and clinicians critically examine how our work can inform or be translated into their research and practice. Future studies are planned to evaluate the effectiveness of MyGoal in achieving better rehabilitation outcomes for adults with chronic conditions.

## Data Availability

The original contributions presented in the study are included in the article/**Supplementary Material**, further inquiries can be directed to the corresponding author.
