# Supplementary material for: Development of a goal setting and goal management system: Intervention Mapping
Source: Front Rehabil Sci. 2024 Jan 8;4:1274191. doi: 10.3389/fresc.2023.1274191 (PMC10801041; doi:10.3389/fresc.2023.1274191)
Supplement: Supplementary file 1 [file Datasheet1.docx]

**Supplementary file 1. MyGoals Activity 4. Make My Goal example**

**Activity 4. Make My Goal**

**Objective:**

- Guide life goal, goal, building block goal formulation
- Guide the evaluation of self-efficacy and positive outcome expectancy levels of goals
- Educate and discuss the benefits of using different goal types
- Educate clients’ health conditions from the biopsychosocial perspective

Based on the previous activity, guide clients to develop life goals, goals, and building block goals. See definitions, examples, and benefits of three goal types in Fig.4.1. Use the prompts/phrases/questions provided in the following pages to educate clients about different goal types.


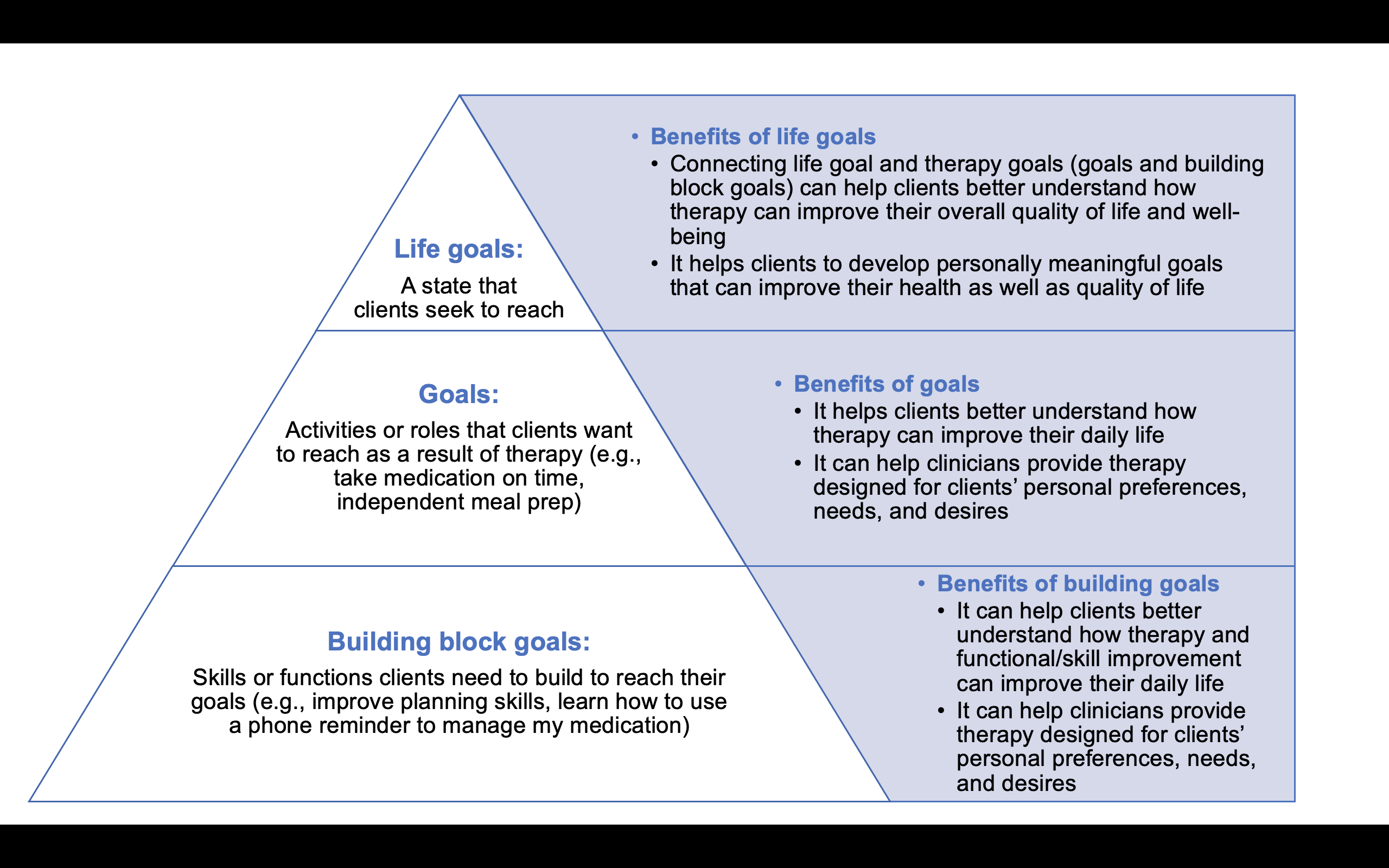


Fig. 4.1 MyGoals pyramid

**MyGoals Goal Pyramid**


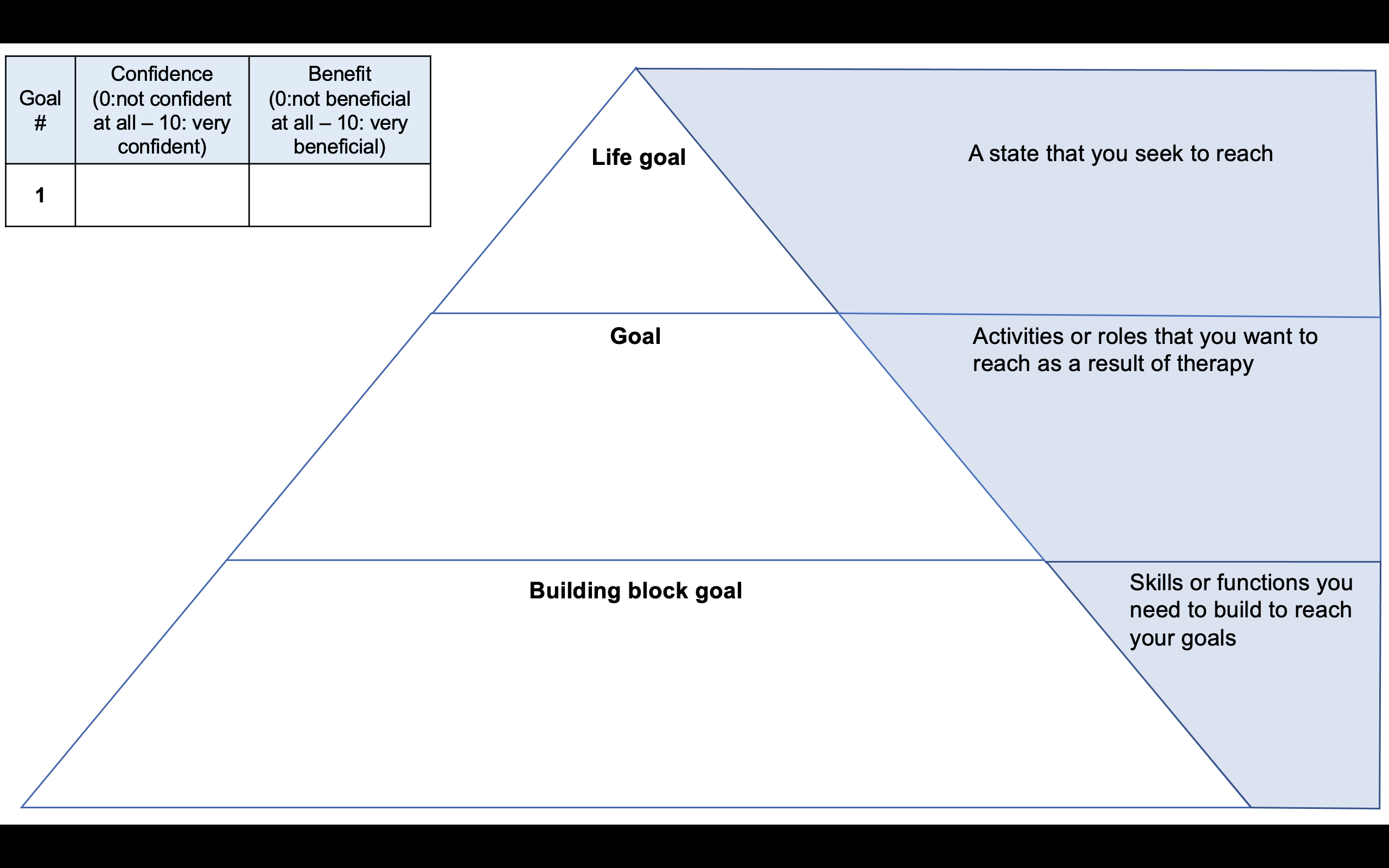


**Activity 4. Make My Goal (cont.)**

1. Provide Find My Goal Sheet to the client.
2. Based on the chosen potential activities or roles, let’s make one goal. Which one do you want to work on?
3. Provide MyGoals Pyramid Sheet to the client.
4. Let’s talk more about your goal to make it more specific. Tell me more about this goal. What do you want to achieve?
5. Write down your goal in the “goal” section of the Pyramid. Make your goal more meaningful and relevant to you using your own words.
6. Given your goal, let’s think about your life goal. A life goal is a state that you seek to reach. It can be about independence, well-being, health, self-image, career, family, relationships, and so on. Life goals can be simple such as *I want to live independently* or as specific as you want.
7. What is your life goal?
8. Write down your life goal in the “life goal” section of the Pyramid.
9. Your life goal may help you see how therapy goals can help you improve your overall quality of life and reach your life goal.
10. Based on your goal, let’s think about your building block goal. Building block goals are skills or functions you need to build to reach your goal. It can be about skills such as planning skills and functions such as walking. What skills or functions do you think can help you to reach your goals? [If clients cannot develop building block goals, provide guidance or examples. These can be explored by using questions about barriers to achieving their goals (e.g., Why is it difficult for you to take medications independently?)]
11. Write down your building block goal in the “building block goal” section of the Pyramid.
12. [Rate confidence to reach each goal] Rate how confident you are in reaching your goal (0: not confident at all – 10: very confident). [If clients have a lower than 7 confidence level, explore what makes them feel less confident. (e.g., Tell me why you rated the confidence 5.) After discussion, if clients are still not confident to reach their goals, consider modifying goals. Do not discourage or force clients to change their goals in this activity. If needed, goals can be revised in Activity 6. It is more important to help clients feel ownership, rather than setting attainable goals.]
13. [Rate perceived potential positive mental, physical, and social outcomes of each goal] Rate how much you may benefit from reaching your goal. Think about the potential positive mental, physical, or social benefits of your goal (0: not beneficial at all – 10: very beneficial). [If clients have a lower than 7 benefit level, explore what makes them see such low benefit (e.g., Tell me about what makes you rate the benefit 5.) Guide clients to see more potential befits of their goals.]
14. [Summarize the goal pyramid] This is your building block goal. [Point to building block goal]. It can help you reach your goal. [Point to the goal] Working towards these goals can help you do your desired activities and reach your life goal [Point to the life goal].

**Supplementary file 2. Mechanisms of actions and parameters for effectiveness incorporated in MyGoals**

| **Mechanisms of action (Applied parameters of effectiveness)** | **How this parameter is incorporated in MyGoals (MyGoals activity number(s) or OT education)** |
| --- | --- |
| Advance organizers (Schematic representation of the intervention contents and guides to the contents) | - Enable clients to understand the concepts of life goals, goals, and building block goals using Goal Pyramid Sheet and verbal education (4) |
| Discussion (Carefully listen to clients to make sure the targeted schemas are activated) | - Educate clinicians about the importance of active listening and ensuring clients’ understanding of the targeted schemas (OT education) - Actively listen to clients and explicitly ask questions to confirm their understanding of the targeted schemas (1,4-6) |
| Elaboration (Personally relevant and easy-to-understand messages with direct instructions) | - Stimulate clients to add meaning to the life goal, goal, and building block goal, guide clients to reflect on what potential outcomes they may have by working towards reaching their life goal, goal, and building block goal, and guide clients to understand that working towards their goals and building block goals can help them reach their life goal (4) - Guide clients to reflect on potential positive outcomes of their plans (5) |
| Individualization (Personal communication tailored to the client’s needs) | - Encourage clients to ask personal questions and provide individualized instructions (1-6) - Provide clients with individualized instructions based on their understandings, and explicitly ask clients whether they understand the intervention concepts (1,4-6) |
| Participation (Clinicians’ willingness to accept clients as active partners in their care; clients with motivation and skills) | - Educate clinicians about the importance of active client engagement (OT education) - Ask open-ended questions and develop easy-to-participate activities using lay language (1-6) - Encourage clients to actively participate in MyGoals activities using verbal education and read out loud the MyGoals summary sheet (1,6) - Guide clients to reflect on their current engagement in activities and their health and environment and share their reflections (2) - Guide clients to come up with and write down activities and roles they want to work on using the Find My Goals Sheet (3) - Guide clients to come up with and write down personally meaningful goals with high confidence and positive outcome expectancy using the Make My Goals Sheet and guide them to realize potential positive outcomes of the developed goals (4) - Guide clients to come up with and write down their facilitators, barriers, and planned actions using My Plan and Progress Sheet, Guide clients to develop personally relevant and confident plans, with high positive outcome expectancy, and guide clients to realize potential positive outcomes of the developed plans (5) - Guide clients to monitor their goal progress using the My Plan and Progress Sheet, guide clients to adjust their goals and/or plans to develop personally meaningful goals and relevant plans with high positive outcome expectancy (6) |
| Self-reevaluation (Provide clients feedback related to affective and cognitive stimulation to appraise their self-image) | - Guide clients to evaluate their cognitive and effective self-image regarding current engagement in activities, potential goal activities, developed goals, plans, goal progress, and goal and/or adjustment and provide feedback to support the appraisal process (2-6) |
| Enactive mastery experiences (A client’s willingness to accept feedback from the clinician) | - Build rapport and trust with clients (1-6) - Guide clients to work on increasingly challenging goals and plans and provide feedback to assure clients that they can achieve those (6) |
| Feedback (Individualized and specific feedback) | - Provide individualized and specific feedback about clients’ goal progress (6) |
| Goal setting (Clients’ commitment to the goal; challenging but attainable goals) | - Guide clients to develop and adjust personally meaningful, challenging, and attainable goals (4,6) - Guide clients to develop and adjust personally relevant and attainable plans (5,6) |
| Implementation intention (Clients’ positive intention) | - Guide clients to develop if-then plans using the planned action/behavior that clients have positive intentions (5) |
| Improving physical and emotional states (Carefully interpret physical and emotional states) | - Guide clients to carefully interpret and manage their emotional states regarding their goal progress (6) |
| Planning coping responses (Identification of barriers and practice coping strategies) | - Guide clients to identify barriers and plan the coping responses to develop coping plans (5) |
| Public commitment (Public announcement) | - Guide clients to announce their goals to clinicians and/or others (4,5) |
| Self-monitoring of behavior (Monitoring of the specific behavior; The monitoring results need to be interpreted and used) | - Guide clients to monitor their goal progress and other goal-related behaviors to interpret and use for the goal progress monitoring (6) |
| Set graded tasks (The difficult level of the final behaviors can be adjusted) | - Guide clients to develop challenging but attainable goals and plans (4-5) |
| Verbal persuasion (Reliable source) | - Reinforce clients that they can reach their plans (5) |
